# Serum urate and pancreatitis: A bidirectional Mendelian randomization study

**DOI:** 10.1097/MD.0000000000046135

**Published:** 2025-11-28

**Authors:** Yanping Li, Jun Liu, Xiaolong Zhao

**Affiliations:** a Geriatric Nursing Department, Chongqing Medical and Pharmaceutical College, Chongqing, China; b Department of Pediatrics, Maternal and Child Health Hospital, Chongqing, China; c Department of Internal Medicine, 956th Hospital of PLA, Linzhi, China.

**Keywords:** acute pancreatitis, causal relationship, chronic pancreatitis, Mendelian randomization, pancreatitis, serum urate

## Abstract

Pancreatitis, a significant global health concern with rising incidence, requires deeper understanding of its modifiable risk factors. This Mendelian randomization study investigated bidirectional causal relationships between serum urate levels and pancreatitis subtypes (acute pancreatitis (AP), alcohol-induced AP, chronic pancreatitis (CP), and alcohol-induced CP) using genome-wide association data from the Global Urate Genetics Consortium and FinnGen Consortium. Through rigorous selection of genetic variants as instrumental variables and application of inverse-variance-weighted methods complemented by Mendelian randomization-Egger and weighted median approaches, we identified a 28% increased AP risk per 1 mg/dL serum urate elevation (OR = 1.28, 95% CI = 1.05–1.57). No significant associations emerged for CP or alcohol-related subtypes. Reverse-direction analysis revealed no causal effects of pancreatitis on urate levels. These findings establish serum urate as a novel risk determinant for AP, highlighting its potential as a therapeutic target. Further investigations should validate these associations across diverse populations and elucidate underlying biological mechanisms.

## 1. Introduction

Pancreatitis is an inflammatory disorder characterized by premature activation of pancreatic enzymes within the gland, leading to autodigestion, edema, hemorrhage, and even necrosis of pancreatic tissue. It is broadly classified into acute pancreatitis (AP) and chronic pancreatitis (CP). Severe AP may rapidly progress to life-threatening complications,^[1]^ while CP often results in progressive pancreatic dysfunction, triggering malabsorption, diabetes mellitus, and other debilitating sequelae that profoundly impair quality of life and impose substantial socioeconomic burdens.^[2]^

Established risk factors for pancreatitis include gallstone disease, alcohol abuse, hyperlipidemia, hypercalcemia, specific medications, and genetic predisposition.^[3–5]^ Despite advances in understanding, the precise pathogenesis remains elusive, likely involving dysregulation of multiple biomarkers and metabolic pathways. Identifying key contributing factors is therefore critical for elucidating disease mechanisms and developing targeted therapies.

Emerging evidence highlights a potential association between elevated serum urate levels and pancreatitis risk. Observational studies suggest hyperuricemia may be a significant risk factor. A study found that for every 1 mg/dL increase in serum uric acid, the risk of pancreatic steatosis increased by 33% (OR = 1.33, 95% CI: 1.01–1.76), and steatosis is closely related to the occurrence of pancreatitis.^[6]^ Furthermore, AP patients exhibit markedly elevated uric acid concentrations compared to healthy controls, with no significant difference between mild and severe cases.^[7]^ In CP, hyperuricemia may exacerbate disease progression and worsen prognosis, as evidenced by a study demonstrating that 61.6% of CP patients developed diabetes mellitus, closely linked to metabolic dysregulation.^[8]^ However, observational designs are inherently limited by residual confounding, reverse causation (e.g., post-pancreatitis alterations in renal function or dietary habits affecting urate metabolism), and high resource demands, thereby undermining causal inference.

Mendelian randomization (MR), an innovative causal inference methodology, leverages genetic variants as instrumental variables (IVs) to emulate randomized controlled trials. Grounded in Mendel Second Law of independent assortment, MR exploits the random allocation of genetic variants at conception, effectively minimizing confounding and reverse causation.^[9,10]^ This approach has been widely adopted to evaluate causal relationships in biomedical research. Nevertheless, no prior MR studies or RCTs have specifically investigated the urate-pancreatitis relationship. To address this gap, we conducted a bidirectional 2-sample MR analysis to elucidate potential causal links between serum urate levels and pancreatitis subtypes, including AP, alcohol-induced AP (AAP), CP, and alcohol-induced CP (ACP), thereby expanding our understanding of the complex interactions between serum urate and pancreatitis.

## 2. Materials and methods

### 2.1. Study design

**Figure 1.**
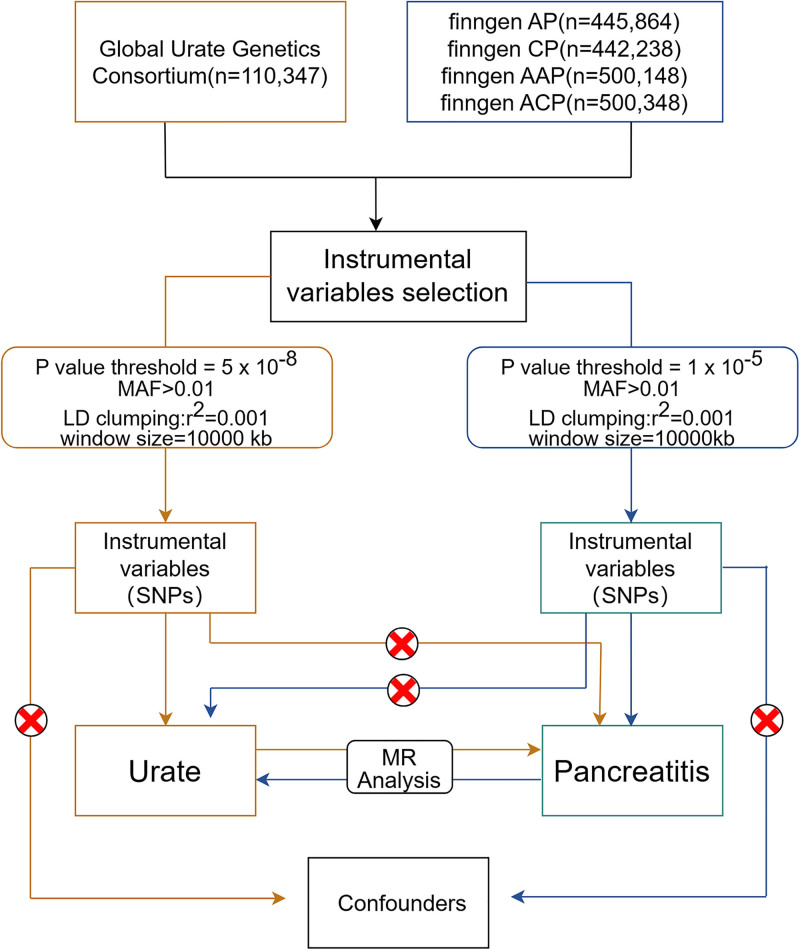
Workflow of the causal inference between urate and pancreatitis. AAP = alcohol-induced acute pancreatitis, ACP = alcohol-induced chronic pancreatitis, AP = acute pancreatitis, CP = chronic pancreatitis, LD = linkage disequilibrium, MAF = minor allele frequency, MR = Mendelian randomization, SNPs = single-nucleotide polymorphisms.

This Mendelian randomization (MR) study investigated bidirectional causal relationships between serum urate levels and pancreatitis subtypes, including AP, alcohol-associated AP (AAP), CP, and alcohol-associated CP (ACP). Genetic variants were employed as IVs, rigorously selected to satisfy 3 core MR assumptions: First, IVs demonstrated strong associations with the exposure. Second, IVs remained independent of potential confounding factors that might distort exposure-outcome relationships. Third, IVs influenced outcomes exclusively through the specified exposure, with no horizontal pleiotropy. The analytical workflow is illustrated in Figure 1. We adhered to the STROBE-MR guidelines (Strengthening the Reporting of MR Studies)^[11]^ to ensure methodological transparency and reporting completeness (Table S1, Supplemental Digital Content, https://links.lww.com/MD/Q768).

### 2.2. Data sources

Summary-level data for serum urate were obtained from the Global Urate Genetics Consortium genome-wide association study (GWAS ID: ieu-a-1055), which measured urate concentrations using primarily the uricase method in 110,347 individuals of European ancestry, encompassing 2450,548 single-nucleotide polymorphisms (SNPs). The mean serum urate concentration ranged from 3.9 to 6.1 mg/dL (median: 5.2 mg/dL). GWAS summary statistics for pancreatitis subtypes were acquired from the FinnGen Consortium (release R12): AP: 8446 cases versus 437,418 controls (https://r12.finngen.fi/pheno/K11_ACUTPANC); CP: 4820 cases versus 437,418 controls (https://r12.finngen.fi/pheno/K11_CHRONPANC); Alcohol-induced AP (AAP):1086 cases versus 499,062 controls (https://r12.finngen.fi/pheno/ALCOPANCACU); Alcohol-induced CP (ACP): 2400 cases versus 497,948 controls (https://r12.finngen.fi/pheno/ALCOPANCCHRON). All participants were of European descent. As this study exclusively utilized publicly accessible summary statistics, no additional ethics approval was required.

### 2.3. Instrumental variables selection

To ensure robust causal inference between urate and pancreatitis, we implemented stringent quality control procedures for IV selection (Fig. 1). Genetic variants associated with exposures were first extracted at genome-wide significance (*P* <5 × 10⁻⁸), with relaxed thresholds (*P* < 1 × 10⁻⁵) applied to pancreatitis GWASs containing ≤3 significant SNPs. SNPs with minor allele frequency <0.01 were excluded, followed by linkage disequilibrium (LD) clumping (window: 10 000 kb; *r*² <0.001) using genotype data from European individuals from phase 3. When target SNPs were unavailable in the dataset, proxy SNPs demonstrating high LD with the index variants (*R*² >0.8) were selected as substitutes. Incompatible alleles and palindromic SNPs with intermediate frequencies were excluded from the MR analysis. Furthermore, SNPs potentially associated with confounding factors or exhibiting pleiotropic effects on outcomes were systematically excluded. IV validity was ultimately confirmed through *F*-statistic evaluation (*F* >10), satisfying the critical threshold for robust causal inference as established in MR methodology.

### 2.4. Statistical analysis

The inverse-variance weighted (IVW) method served as the primary approach for causal effect estimation, supplemented by other methods including MR-Egger, weighted median, simple mode, and weighted mode. As a meta-analytic technique, IVW combines Wald ratio estimates from individual genetic variants to derive consistent causal estimates under the assumption that all IVs are valid^[12]^; However, IVW estimates may be biased in the presence of horizontal pleiotropy. MR-Egger provides causal effect estimates even when all SNPs exhibit directional pleiotropy,^[13]^ though its utility is limited by sensitivity to outliers and reduced statistical power due to wider confidence intervals. The weighted median requires ≥ 50% valid IVs,^[14]^ while the simple mode method employs an unweighted model of the empirical density function for causal estimates.^[15]^ The weighted mode method demonstrates robustness when the largest cluster of similar causal estimates originates from valid IVs.^[16]^ Heterogeneity among genetic variants was assessed using Cochran Q test, with random-effects IVW models applied when *P* <.05. Pleiotropy was evaluated through MR-Egger intercept analysis and the MR-PRESSO global test. Leave-one-out sensitivity analysis identified influential SNPs and verified result stability.^[15]^ To account for multiple comparisons, we applied Bonferroni correction across all independent analyses. A total of 8 tests were conducted (4 forward and 4 reverse MR analyses). Thus, the Bonferroni-adjusted significance threshold was calculated as 0.05/8 = 0.00625. Only results with *P*-value < 0.00625 were considered statistically significant. All analyses were conducted using R v4.4.2 with the TwoSampleMR (version 0.6.11) and MR-PRESSO (version 1.0) packages.

## 3. Results

### 3.1. Effects of serum urate on pancreatitis

#### 3.1.1. Instrumental variable selection

Initial screening identified 24 SNPs significantly associated with serum urate levels at genome-wide significance (*P* < 5 × 10⁻⁸). The rs1260326 variant (GCKR gene) was subsequently excluded due to its established associations with fasting glucose and triglyceride metabolism (PMIDs: 18556336, 18678614, 19643913, 20081858), potentially violating the independence assumption. Final analysis incorporated 23 SNPs fulfilling all 3 core MR assumptions, each demonstrating strong instrument strength (*F*-statistic >10; Table S2, Supplemental Digital Content, https://links.lww.com/MD/Q769).

#### 3.1.2. Association between urate and AP

**Figure 2.**
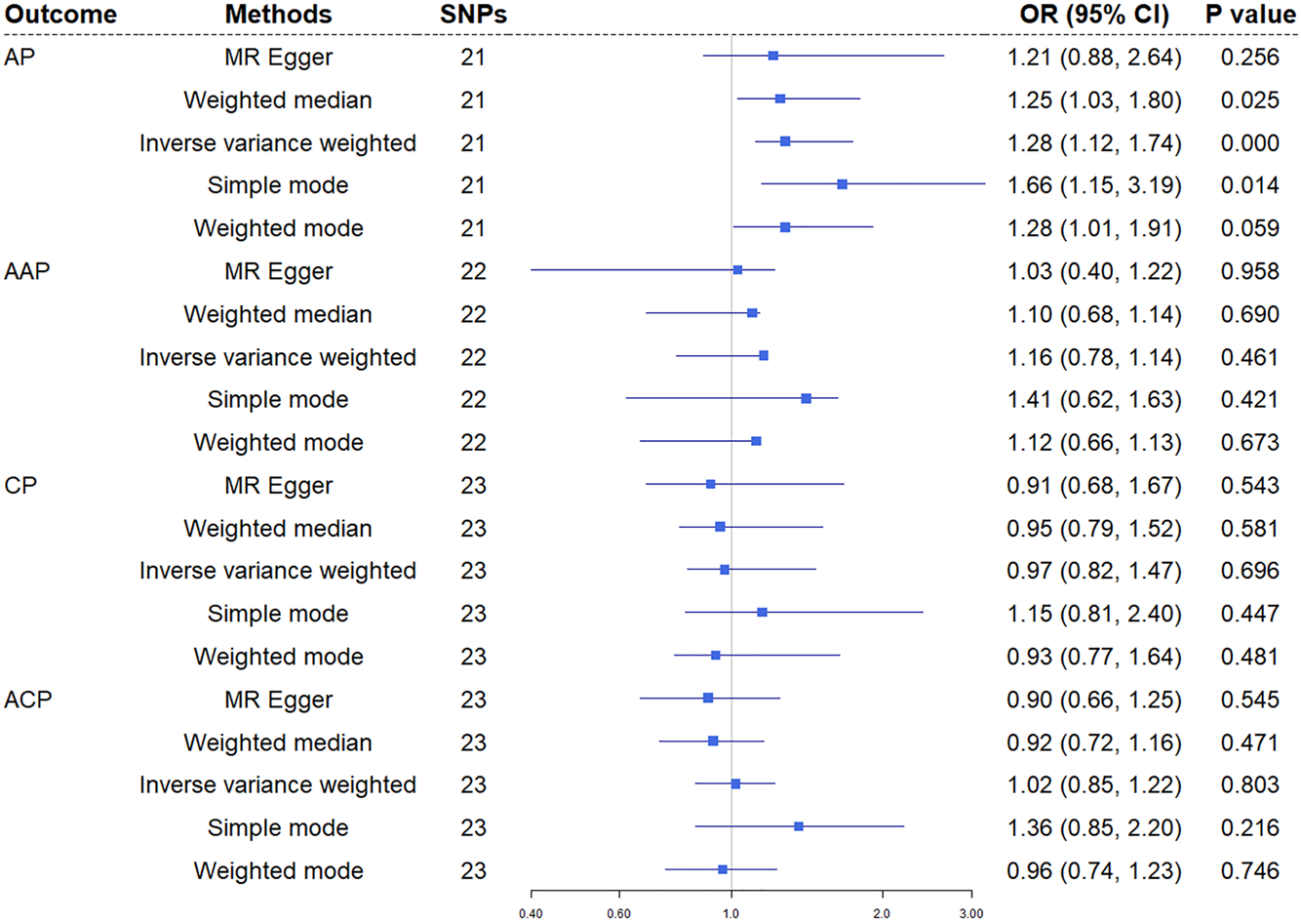
Mendelian randomization results for association of urate on pancreatitis. AAP = alcohol-induced acute pancreatitis, ACP = alcohol-induced chronic pancreatitis, AP = acute pancreatitis, CI = confidence interval, CP = chronic pancreatitis, MR = Mendelian randomization, OR = odds ratio, SNPs = single-nucleotide polymorphisms.

Initial IVW analysis revealed no significant association between serum urate levels and AP risk (OR = 1.104, 95% CI = 0.964–1.264, *P* = .151). Cochran Q test indicated substantial heterogeneity (Q = 38.62, *P* = .026), while MR-PRESSO global testing identified significant pleiotropy (global test *P* = .013). Following exclusion of 2 outlier SNPs (rs11722228 and rs10761587), IVW analysis demonstrated a 28.1% increased AP risk per standard deviation elevation in serum urate (OR = 1.281, 95% CI = 1.05–1.57, *P* = 4.59 × 10⁻⁴) (Fig. 2). Post-exclusion analyses resolved heterogeneity (*P* = .221) and showed no evidence of horizontal pleiotropy via MR-Egger intercept test (*P* = .714) (Table 1).

**Table 1:** Evaluation of heterogeneity and directional pleiotropy.

Exposure	Outcome	Heterogeneity	Pleiotropy	
		Cochran Q (IVW) *P*-value	MR-Egger Intercept *P*-value	MR-PRESSO Global test *P*-value
Urate	AP	.221	.714	.051
Urate	AAP	.026	.360	.021
Urate	CP	.032	.632	.033
Urate	ACP	.501	.372	.212
AP	Urate	.695	.657	.563
AAP	Urate	.281	.927	.306
CP	Urate	.789	.603	.865
ACP	Urate	.869	.621	.823

AAP = alcohol-induced acute pancreatitis, ACP = alcohol- induced chronic pancreatitis, AP = acute pancreatitis, CP = chronic pancreatitis, IVW = inverse-variance-weighted, MR = Mendelian randomization.

#### 3.1.3. Association between urate and AAP

In the AAP subgroup, initial IVW analysis showed no significant association between serum urate levels and AAP (OR = 1.012, 95% CI = 0.738–1.389, *P* = .940). MR-PRESSO identified rs11722228 as a potential outlier, with post-exclusion heterogeneity testing revealing persistent heterogeneity (*P* = .026) (Table 1). Subsequent random-effects IVW analysis maintained nonsignificance (OR = 1.163, 95% CI = 0.779–1.736, *P* = .461), a finding consistently supported by supplementary methods including weighted median, MR-Egger regression, simple mode, and weighted mode analyses (Fig. 2).

#### 3.1.4. Association between urate and CP

Random-effects IVW analysis revealed no significant association between serum urate levels and CP risk (OR = 0.968, 95% CI = 0.823–1.139, *P* = .696), with significant residual heterogeneity observed (*P* = .032) (Table 1). MR-PRESSO global testing confirmed no outliers requiring exclusion. Results from 4 supplementary methods (weighted median, MR-Egger, simple mode, weighted mode) consistently showed no statistically significant relationships (all *P* > .05), reinforcing the stability and reliability of these findings (Fig. 2).

#### 3.1.5. Association between urate and ACP

In the ACP subgroup, IVW analysis demonstrated no significant association between serum urate levels and ACP (OR = 1.023, 95% CI = 0.825–1.224, *P* = .803) (Fig. 2). Both Cochran Q test (*P* = .501) and MR-Egger intercept analysis (*P* = .372) showed no evidence of heterogeneity or horizontal pleiotropy, confirming analytical robustness (Table 1).

### 3.2. Effects of pancreatitis on serum urate

#### 3.2.1. Instrumental variable selection

In reverse-direction MR analysis, initial screening of AP GWAS data identified 14 candidate IVs. The rs11887534 variant was excluded due to its established association with sitosterolemia (PMID: 22898925), a monogenic disorder linked to familial hypercholesterolemia. Final analyses incorporated 13 SNPs for AP, 8 SNPs for alcohol-associated AP (AAP), 14 SNPs for CP, and 8 SNPs for alcohol-associated CP (ACP). All retained instruments demonstrated strong validity (*F*-statistic >10; Table S3, Supplemental Digital Content, https://links.lww.com/MD/Q769).

#### 3.2.2. Effects of pancreatitis on serum urate levels

**Figure 3.**
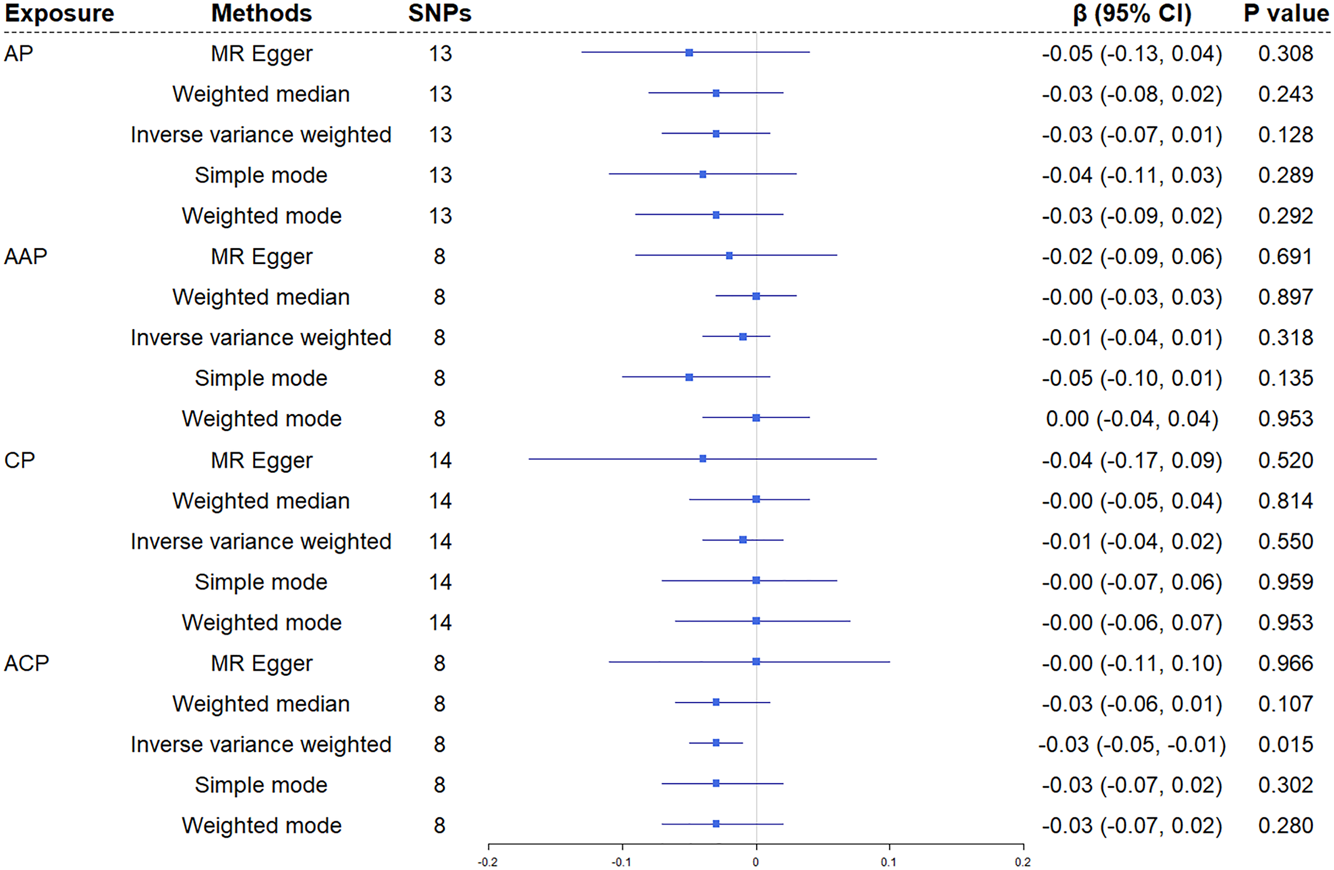
Mendelian randomization results for association of pancreatitis on urate. AAP = alcohol-induced acute pancreatitis, ACP = alcohol-induced chronic pancreatitis, AP = acute pancreatitis, CI = confidence interval, CP = chronic pancreatitis, MR = Mendelian randomization, SNPs = single-nucleotide polymorphisms.

Reverse MR analysis revealed no significant causal associations between any pancreatitis subtypes and serum urate levels (Fig. 3), with no evidence of heterogeneity (Cochran *Q P* >.05) or horizontal pleiotropy (MR-Egger intercept *P* >.05) (Table 1). Notably, genetically predicted CP showed nominal association with lower urate levels in IVW analysis (β = −0.030, 95% CI = −0.054–0.006, *P* = .015), though this finding did not survive Bonferroni correction (*P* <.006 required) and was unsupported by 4 supplementary methods.

### 3.3. Visualization of sensitivity analyses

Forest plots demonstrated individual SNP effect estimates with 95% confidence intervals (Figs. S1–2, Supplemental Digital Content, https://links.lww.com/MD/Q767), while scatter plots confirmed linear associations between genetic instruments and exposure-outcome relationships (Figs. S3–4, Supplemental Digital Content, https://links.lww.com/MD/Q767). Funnel plots revealed symmetrical distribution patterns (Figs. S5–6, Supplemental Digital Content, https://links.lww.com/MD/Q767), suggesting consistency in instrument effect sizes and supporting the absence of substantial directional pleiotropy. Leave-one-out sensitivity analyses indicated no substantial alterations in causal estimates upon sequential SNP exclusion (Figs. S7–8, Supplemental Digital Content, https://links.lww.com/MD/Q767), collectively verifying the robustness of these findings.

## 4. Discussion

Our genome-wide analysis leveraging large-scale GWAS datasets demonstrates a novel positive association between genetically elevated serum urate levels and AP risk. In contrast, no significant associations were observed for CP or its alcohol-related subtypes. The unidirectionality of this relationship is further supported by reverse-direction MR analyses showing null effects of pancreatitis liability on urate homeostasis.

These findings extend previous observational evidence by establishing temporality and minimizing confounding through MR. Clinically, a prospective study of 205 AP patients revealed significantly elevated serum urate levels compared to healthy controls, with weak correlation to triglyceride levels.^[7]^ A large-cohort study further identified elevated serum urate as an independent risk factor for AP, particularly prominent in heavy drinkers.^[17]^ Notably, research on chronic kidney disease patients undergoing antituberculosis therapy demonstrated that drug-induced reduction of urate excretion raises the risk of AP,^[18]^ highlighting the clinical relevance of urate dysregulation. Experimental evidence from animal models supports these clinical observations. In rabbits subjected to self-bile injection, significant increases in plasma xanthine and urate were observed within 12 hours, suggesting a potential role of purine metabolism dysregulation in acute pancreatic necrosis.^[19]^ Mechanistically, the role of urate in AP progression may involve oxidative stress and inflammatory pathways. High-concentration urate induces reactive oxygen species production, leading to cell damage and tissue inflammation.^[20]^ Furthermore, urate enhances local and systemic inflammation by activating the NLRP3 inflammasome, a key driver of AP severity.^[21]^ Synergistically, these pathways impair pancreatic cell function and exacerbate injury.

While urate appears mechanistically involved in AP, our study found no significant association between serum urate levels and CP. This absence of correlation likely reflects the distinct pathophysiological landscape of CP, characterized by multifactorial metabolic dysregulation and self-perpetuating inflammatory pathways.^[22]^ Our findings also demonstrated no correlation between urate and alcohol-induced pancreatitis. The pathogenesis of alcoholic pancreatitis primarily stems from chronic ethanol consumption triggering premature zymogen activation in pancreatic acinar cells, oxidative stress responses, and subsequent chronic inflammation with fibrotic remodeling. While hyperuricemia frequently coexists in patients with alcoholic pancreatitis^[23]^, its potential causal relationship with pancreatitis development may be confounded or obscured by concurrent metabolic disturbances.^[24]^ Furthermore, interindividual variations in alcohol metabolism capacity might modulate the strength of urate-alcoholic pancreatitis associations.^[25]^ Notably, reverse MR analysis showed no significant causal effects of pancreatitis on circulating urate concentrations, substantiating the hypothesis that urate acts specifically as a risk determinant for AP rather than being consequentially modulated by pancreatic inflammation.

Our study has several limitations that should be acknowledged. Firstly, constrained by available database resources, we were unable to acquire biliary pancreatitis-related data, thus precluding analysis of potential urate-biliary pancreatitis associations. Notably, our analysis revealed a significant association between urate levels and AP overall, yet no such relationship emerged specifically for alcohol-associated AP. Given that biliary stones and alcohol account for 70% to 80% of all AP etiologies,^[26]^ this discrepancy strongly suggests that the observed urate-pancreatitis association may be primarily driven by biliary etiology. Future studies should prioritize obtaining biliary-specific datasets to comprehensively evaluate urate’s pathophysiological role across distinct pancreatitis subtypes. Secondly, the limited sample size of alcohol-AAP in current GWAS repositories (n = 1086 cases) likely resulted in insufficient statistical power. Third, our reverse MR analysis revealed a marginal association between CP and urate levels that did not survive multiple testing correction. This borderline signal warrants replication in larger cohorts to explore potential bidirectional mechanisms. Finally, our European-focused GWAS data may limit result generalizability, as genetic and environmental factors influencing urate-pancreatitis relationships can differ across ethnic groups. This population bias highlights the need for diverse cohort validation.

## 5. Conclusion

Our MR study reveals a significant positive association between elevated serum urate levels and AP risk. No substantial links were found between serum urate and CP or alcohol-related subtypes. Reverse MR analyses showed no significant causal effects of pancreatitis on serum urate levels. This study highlights serum urate as a potential biomarker and therapeutic target for AP, offering valuable insights for clinical management. Future research should focus on validating these findings in diverse populations and exploring the underlying biological mechanisms.

## Acknowledgments

Supporting data and materials for serum urate were obtained from the Global Urate Genetics Consortium (GUGC) genome-wide association study (GWAS ID: ieu-a-1055). The GWAS summary statistics for pancreatitis subtypes were acquired from the FinnGen Consortium (release R12). We thank all participants and investigators for contributing to the GWAS data.

## Author contributions

**Conceptualization:** Xiaolong Zhao.

**Data curation:** Yanping Li, Jun Liu.

**Formal analysis:** Yanping Li.

**Methodology:** Xiaolong Zhao, Yanping Li.

**Supervision:** Xiaolong Zhao, Jun Liu.

**Writing – original draft:** Yanping Li.

**Writing – review & editing:** Xiaolong Zhao, Jun Liu.

## Supplementary Material






