# Supplementary material for: Serum urate and pancreatitis: A bidirectional Mendelian randomization study
Source: Medicine (Baltimore). 2025 Nov 28;104(48):e46135. doi: 10.1097/MD.0000000000046135 (PMC12662395; doi:10.1097/MD.0000000000046135)

Supplementary Fig 1 Single SNP effect combination forest plot between urate and pancreatitis. (A) Single SNP effect combination forest plot of the causal relationships between urate and AP. (B) Single SNP effect combination forest plot of the causal relationships between urate and AAP. (C) Single SNP effect combination forest plot of the causal relationships between urate and CP. (D) Single SNP effect combination forest plot of the causal relationships between urate and ACP.

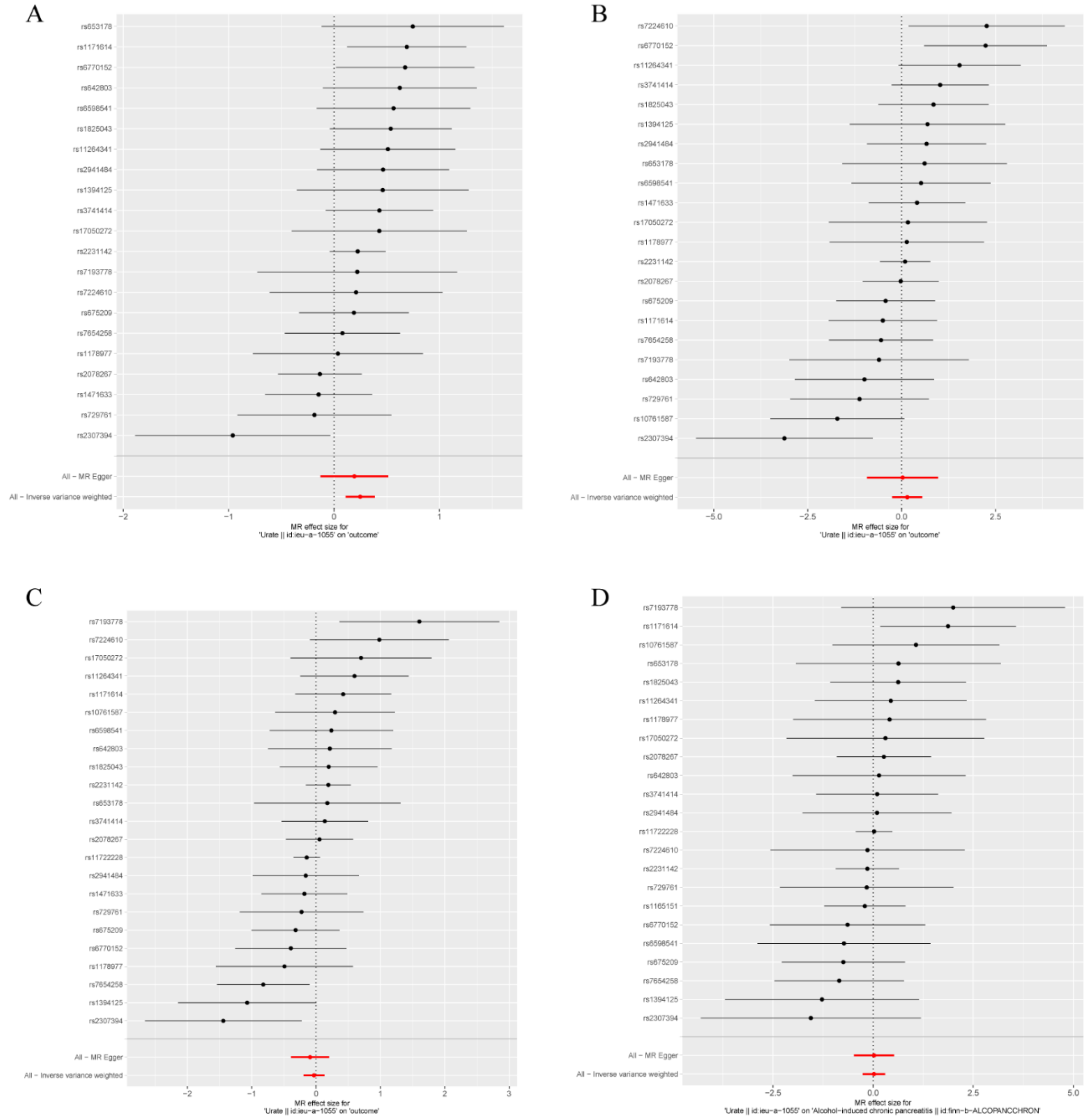

Supplementary Fig 2 Single SNP effect combination forest plot between pancreatitis and urate. (A) Single SNP effect combination forest plot of the causal relationships between AP and urate. (B) Single SNP effect combination forest plot of the causal relationships between AAP and urate. (C) Single SNP effect combination forest plot of the causal relationships between CP and urate. (D) Single SNP effect combination forest plot of the causal relationships between ACP and urate.

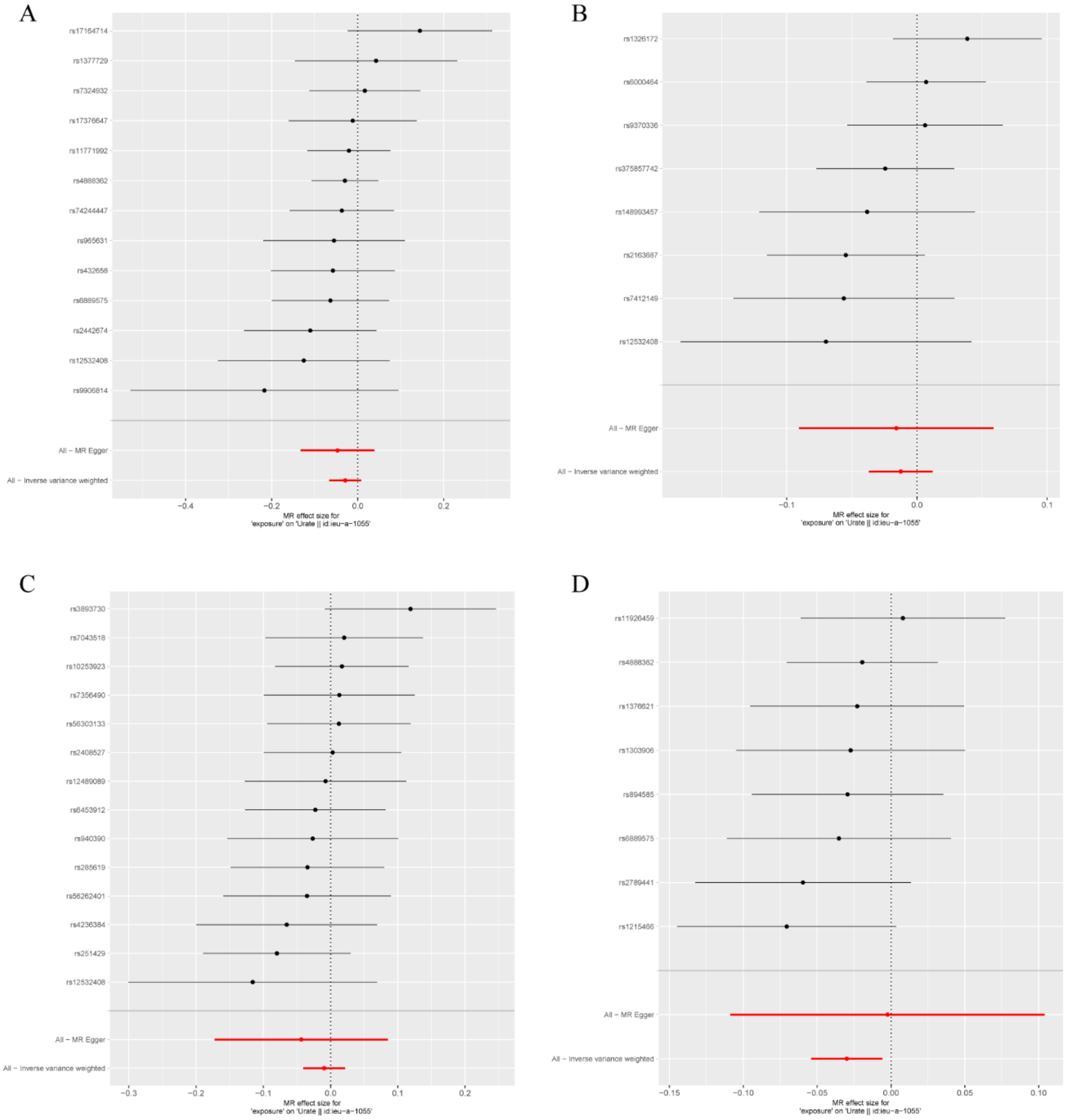

Supplementary Fig 3 Scatter plot of the effect of urate on pancreatitis. (A) Scatter plot of the effect of urate on AP. (B) Scatter plot of the effect of urate on AAP. (C) Scatter plot of the effect of urate on CP. (D) Scatter plot of the effect of urate on ACP.

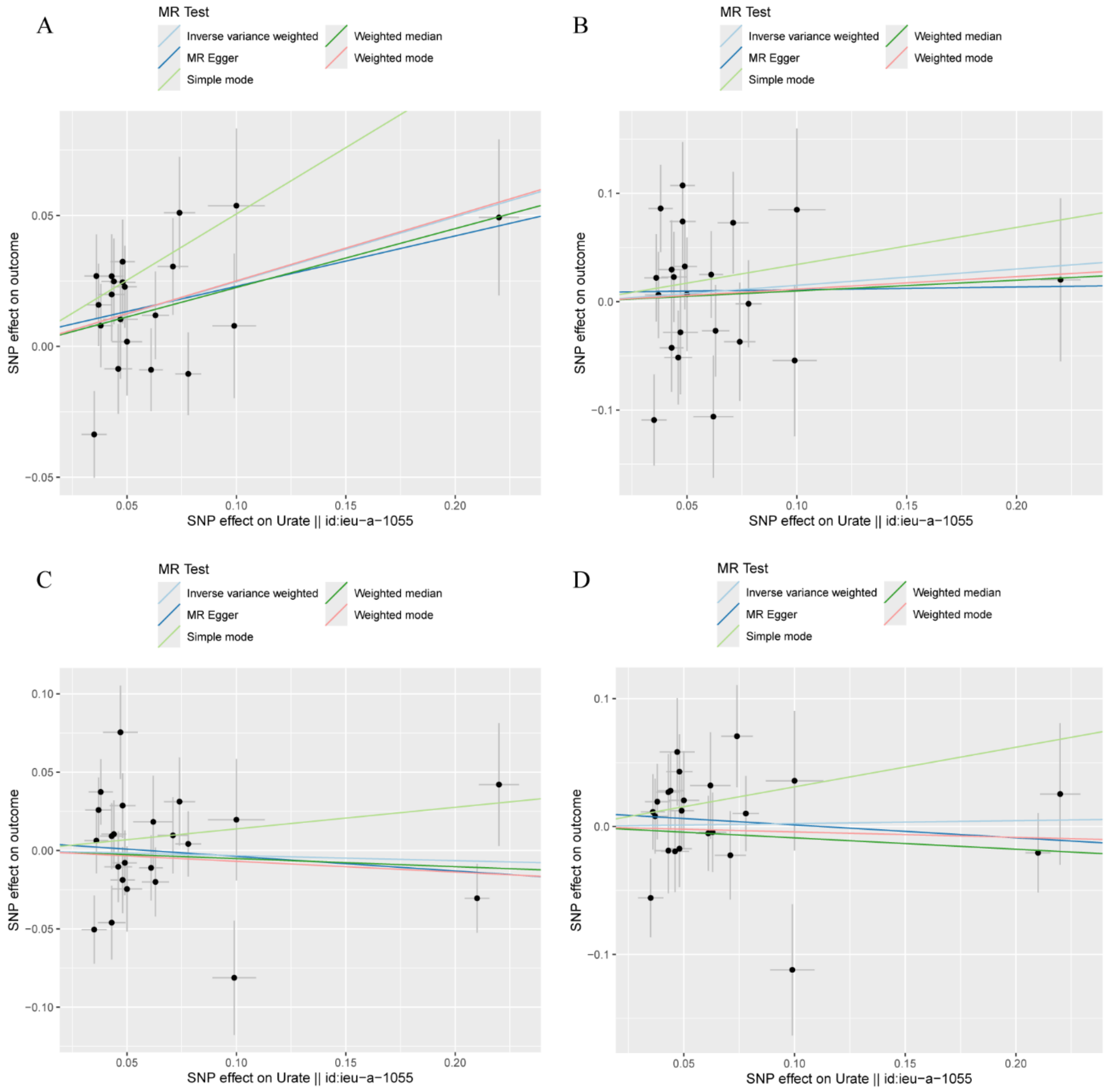

Supplementary Fig 4 Scatter plot of the effect of pancreatitis on urate. (A) Scatter plot of the effect of AP on urate. (B) Scatter plot of the effect of AAP on urate. (C) Scatter plot of the effect of CP on urate. (D) Scatter plot of the effect of ACP on urate.

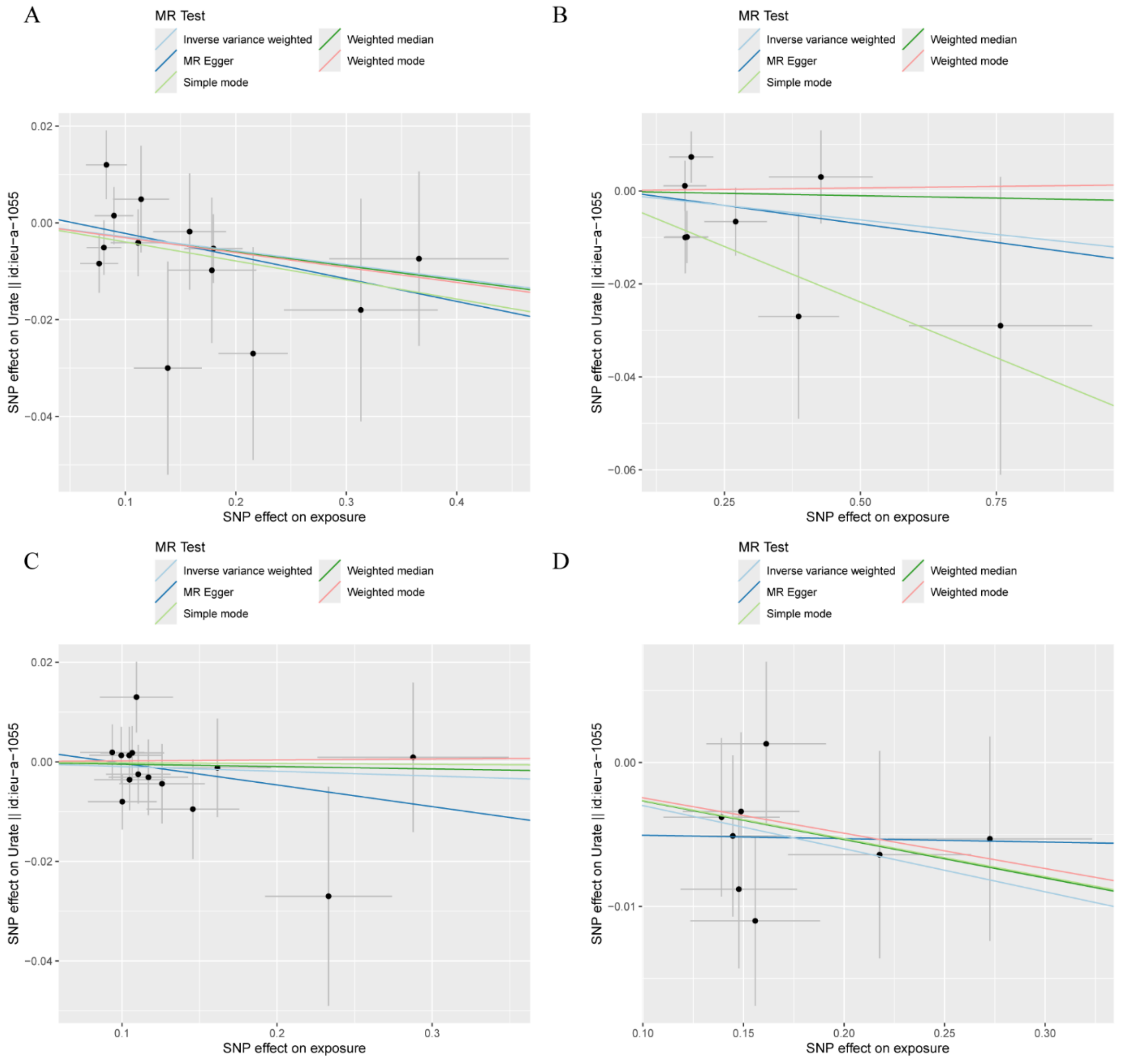

Supplementary Fig 5 Funnel plot of Mendelian randomization analysis for the association between urate and pancreatitis. (A) Funnel plot of the association between urate and AP. (B) Funnel plot of the association between urate and AAP. (C) Funnel plot of the association between urate and CP. (D) Funnel plot of the association between urate and ACP.

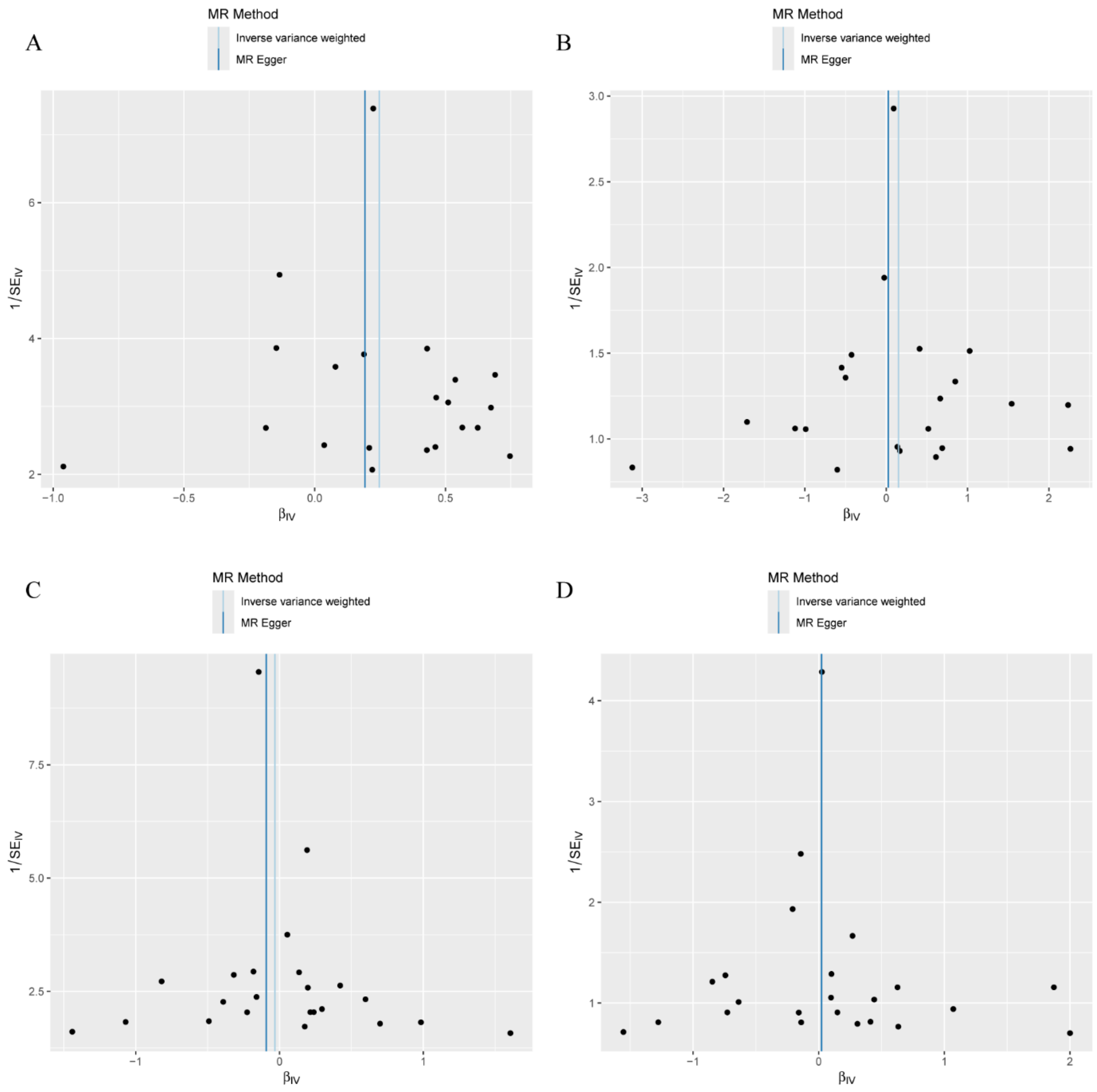

Supplementary Fig 6 Funnel plot of Mendelian randomization analysis for the association between pancreatitis and urate. (A) Funnel plot of the association between AP and urate. (B) Funnel plot of the association between AAP and urate. (C) Funnel plot of the association between CP and urate. (D) Funnel plot of the association between ACP and urate.

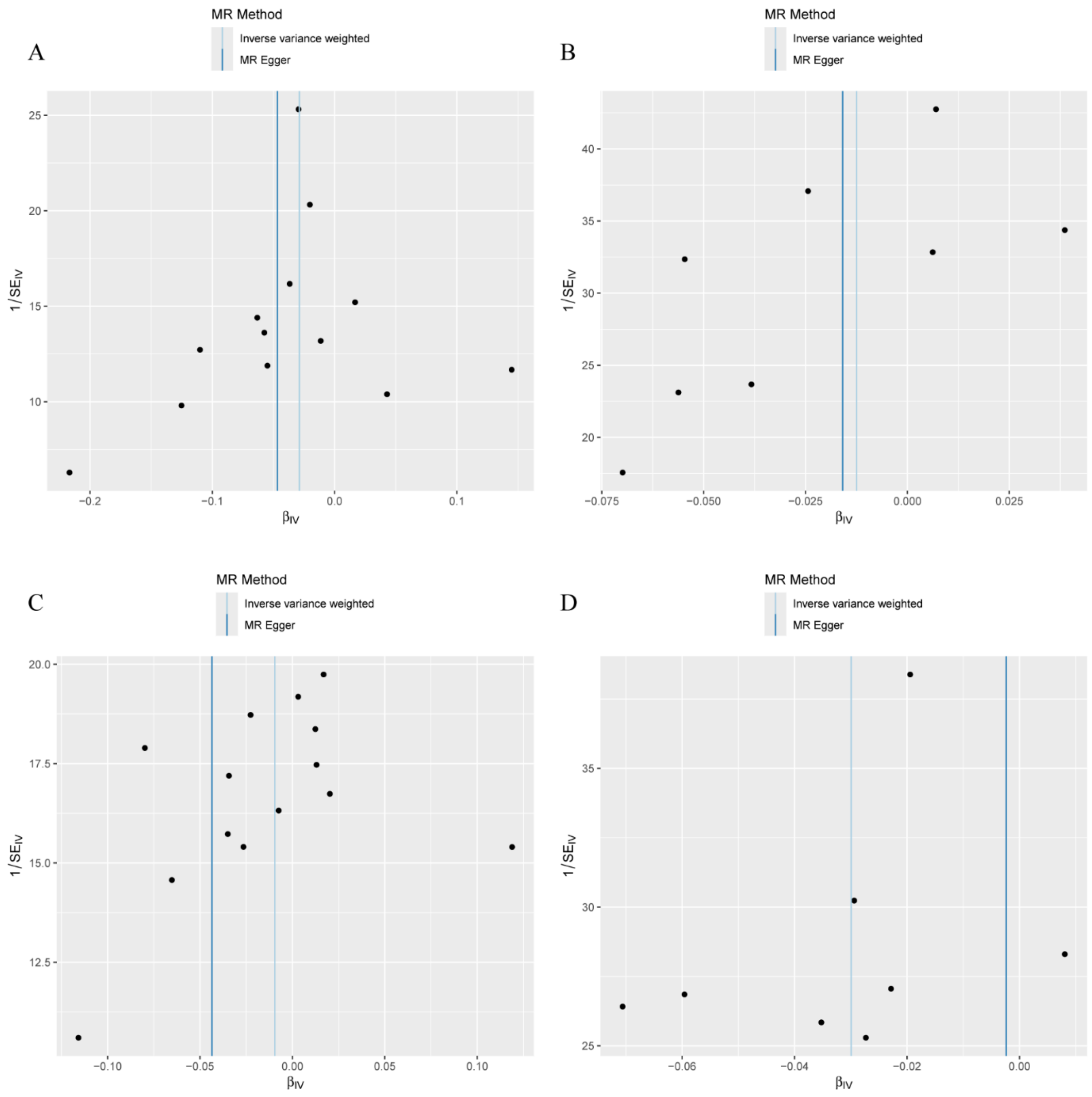

Supplementary Fig 7 Leave-one-out sensitivity analysis for the causal association between urate and pancreatitis. (A) Leave-one-out sensitivity analysis for the causal association between urate and AP. (B) Leave-one-out sensitivity analysis for the causal association between urate and AAP. (C) Leave-one-out sensitivity analysis for the causal association between urate and CP. (D) Leave-one-out sensitivity analysis for the causal association between urate and ACP.

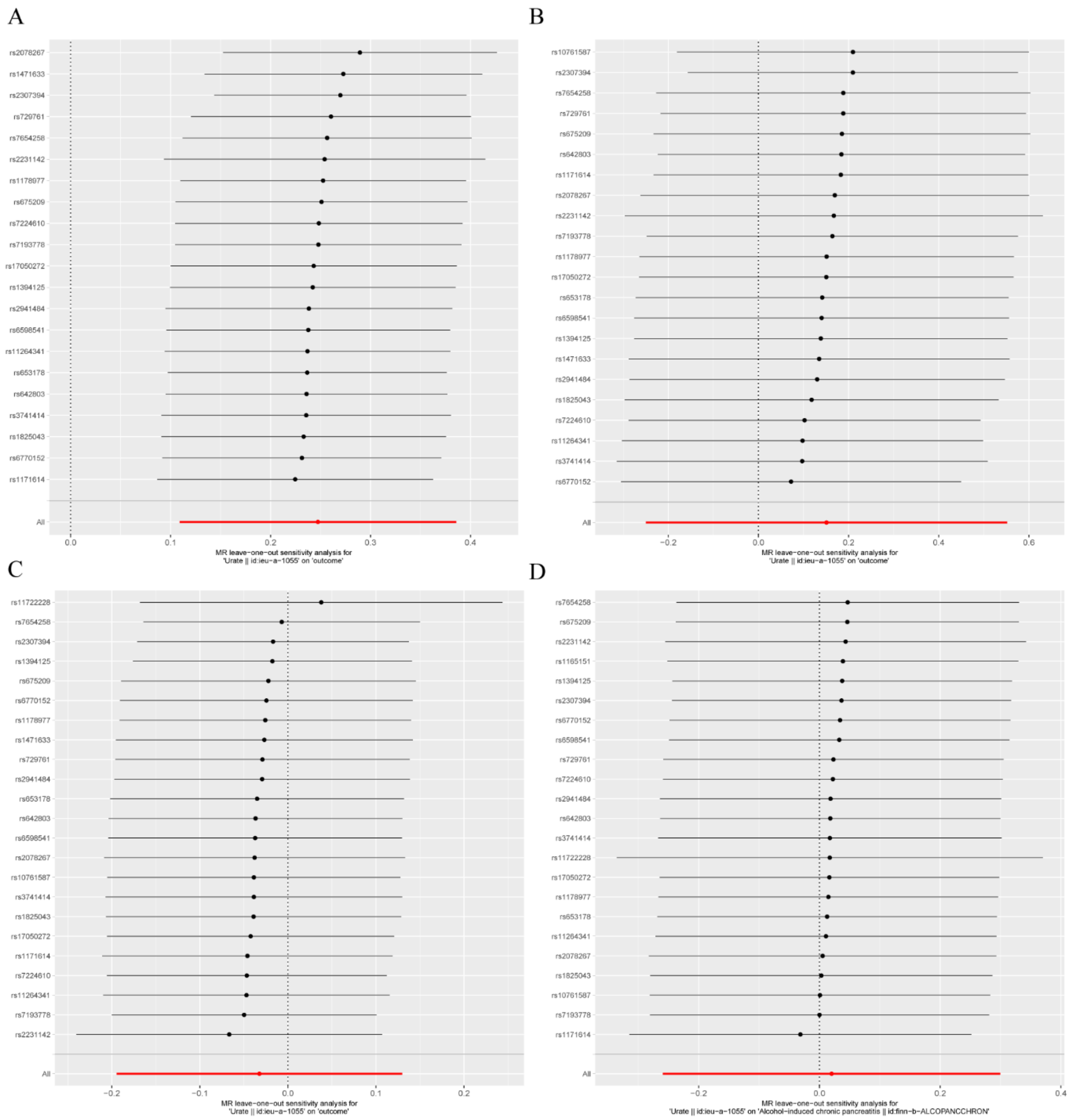

Supplementary Fig 8 Leave-one-out sensitivity analysis for the causal association between pancreatitis and urate. (A) Leave-one-out sensitivity analysis for the causal association between AP and urate. (B) Leave-one-out sensitivity analysis for the causal association between AAP and urate. (C) Leave-one-out sensitivity analysis for the causal association between CP and urate. (D) Leave-one-out sensitivity analysis for the causal association between ACP and urate.

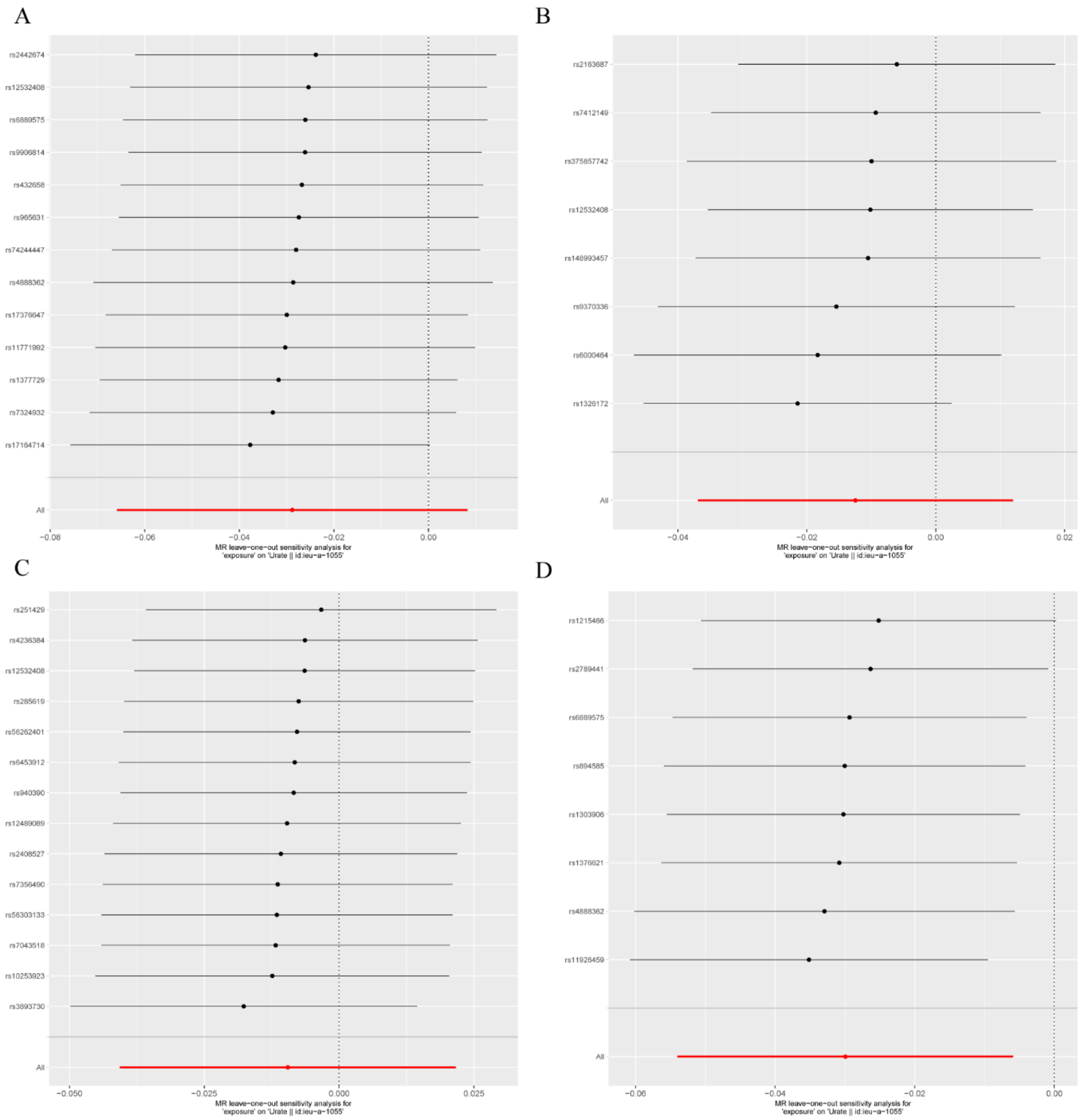

Supplement: Supplementary file 3 [file medi-104-e46135-s003.pdf]
